# miR-210-3p enriched extracellular vesicles from hypoxic neuroblastoma cells stimulate migration and invasion of target cells

**DOI:** 10.1186/s13578-023-01045-z

**Published:** 2023-05-18

**Authors:** Pina Fusco, Anna Fietta, Maria Rosaria Esposito, Luca Zanella, Sara Micheli, Angelica Bastianello, Lorenzo Bova, Giulia Borile, Giuseppe Germano, Elisa Cimetta

**Affiliations:** 1 Department of Industrial Engineering (DII), University of Padua, Via Marzolo 9, 35131 Padua, Italy; 2 Fondazione Istituto Di Ricerca Pediatrica Città Della Speranza (IRP), Corso Stati Uniti 4, 35127 Padua, Italy

**Keywords:** Neuroblastoma, Extracellular vesicles, miRNA, Hypoxia, Metastasis

## Abstract

**Background:**

Tumor hypoxia stimulates release of extracellular vesicles (EVs) that facilitate short- and long-range intercellular communication and metastatization. Albeit hypoxia and EVs release are known features of Neuroblastoma (NB), a metastasis-prone childhood malignancy of the sympathetic nervous system, whether hypoxic EVs can facilitate NB dissemination is unclear.

**Methods:**

Here we isolated and characterized EVs from normoxic and hypoxic NB cell culture supernatants and performed microRNA (miRNA) cargo analysis to identify key mediators of EVs biological effects. We then validated if EVs promote pro-metastatic features both in vitro and in an in vivo zebrafish model.

**Results:**

EVs from NB cells cultured at different oxygen tensions did not differ for type and abundance of surface markers nor for biophysical properties. However, EVs derived from hypoxic NB cells (hEVs) were more potent than their normoxic counterpart in inducing NB cells migration and colony formation. miR-210-3p was the most abundant miRNA in the cargo of hEVs; mechanistically, overexpression of miR-210-3p in normoxic EVs conferred them pro-metastatic features, whereas miR-210-3p silencing suppressed the metastatic ability of hypoxic EVs both in vitro and in vivo.

**Conclusion:**

Our data identify a role for hypoxic EVs and their miR-210-3p cargo enrichment in the cellular and microenvironmental changes favoring NB dissemination.

**Supplementary Information:**

The online version contains supplementary material available at 10.1186/s13578-023-01045-z.

## Introduction

Neuroblastoma (NB) is a pediatric tumor derived from the sympathoadrenal lineage of neural crest progenitor cells and represents the most common malignancy in early childhood [1, 2]. NB is biologically heterogeneous, and its prognosis variable [3]. Over half of NB patients classified as high-risk, already display metastatic disease at diagnosis. Metastases are primarily located in the bone marrow, but also colonize bone, lymph nodes, liver, and skin. Amplification of the MYC family member *MYCN* oncogene is found in ~ 25% of all NB cases and represents the genetic lesion most consistently associated with a poor outcome [4]. Currently, amplification of MYCN remains the best-characterized genetic marker that is still used to stratify risk. MYCN amplification status strongly correlates with higher tumor aggression, specifically with invasive and metastatic behavior. Despite aggressive treatment strategies, the prognosis of high-risk patients with disseminated NB is grim, with an approximate 40% overall 5 years survival rate [5]. NB, like other solid tumors, is characterized by hypoxic areas leading to tissue necrosis and neovascularization [6]. Hypoxia favors NB dedifferentiation towards a more immature cell phenotype and is associated with NB metastasis [7]. Interestingly, when NB cells are exposed to hypoxic environments, they prime normoxic cells located in their proximity to a pro-metastatic and more aggressive behavior, suggesting that factors released from hypoxic cells can act paracrinally to modify the overall features of the primary tumor [8]. Intermittent hypoxia, or hypoxia followed by re-oxygenation, is a common phenomenon in solid tumors including NB, and impacts on the outcome of therapies [9]. Transient hypoxia can be tied to the presence of aberrant vessels, frequently sprouted to support tumor growth, which tend to have impaired flow impeding a steady oxygen transport to the neighboring tissues [10].

Molecularly, hypoxia stimulates hypoxia-inducible factors (HIFs) in NB cells directly exposed to low oxygen tension, with far reaching effects on angiogenesis, metabolic reprogramming, extracellular matrix remodeling, epithelial-mesenchymal transition (EMT), motility, invasion, metastasis, cancer stem cell maintenance, immune evasion, and resistance to chemo and radiation therapy [11]. In addition, extracellular vesicles (EVs) secreted from tumor cells are increasingly recognized as mediators of the crosstalk between tumor cells and the microenvironment, both locally and at distant recipient sites [12, 13]. EVs are categorized mostly based on their size in small EVs (sEVs, < 100 nm or < 200 nm) and medium/large EVs (m/lEVs, > 200 nm) [14, 15]. EVs can reach target cells and deliver their content to induce functional responses and promote phenotypic changes, with the potential to affect physiological or pathological statuses [16]. The cargo of EVs includes growth factors, proteins, RNAs, miRNAs, and lipids, reflecting the function and molecular signatures of the host cell [17]. EVs surface is decorated with proteins that facilitate the selective targeting and uptake by recipient cells, contributing to the formation of pre-metastatic niches permissive for the influx, engraftment, and survival of incoming metastatic cells [12, 18]. However, whether EVs derived from NB cells cultured under low oxygen tensions (hEVs) can contribute to the increased aggressiveness is still unclear. We therefore investigated whether hEVs induce pro-metastatic phenotypes in normoxic target cells, mimicking in vitro the primary tumor niche.

Despite comparable structural and surface characteristics, EVs released by two NB cell lines cultured in hypoxic conditions are more potent than their normoxic counterparts in increasing migration and colony forming ability of naïve NB cells by triggering expression of genes involved in EMT and invasiveness. We identified miR-210-3p as the most upregulated miRNA in hypoxic NB-derived EVs, and its modulation strongly affected the migration and invasiveness of NB cells both in vitro and in vivo. Overall, our data support a role for EVs from hypoxic NB cells and their miR-210-3p cargo enrichment in promoting features of NB aggressiveness.

## Methods

### Cell culture

NB SK-N-AS and SK-N-DZ cell lines (ATCC, CRL 2137; ATCC, CRL 2149) were cultured in DMEM supplemented with 10% FBS (both ATCC), 2 mM glutamine, 100 U/ml penicillin/streptomycin (Life Technologies) and 1 × MEM non-Essential Amino Acids (Biowest) at 37 °C in 5% CO_2_. For hypoxic studies, cells were maintained at 37 °C with 5% CO_2_ and 1.5% O_2_ in Whitley H35 Hypoxystation (Don Whitley Scientific Limited).

### Transient Transfection of miRNA

mirVana miR-210-3p miRNA mimic (MC10516), mirVana miRNA inhibitor (MH10516) and negative control (miR-NC) were purchased from Ambion (Thermo Fisher Scientific). Forty-eighth hours before transfection, 2.5 × 10^6^ SK-N-AS and SK-N-DZ cells were seeded in 10 ml of complete growth medium for each condition to reach 60–80% confluence at the time of the experiment. Cells were reverse-transfected using Lipofectamine RNAiMAX (Invitrogen, 13778–075) in OPTI-MEM reduced serum medium (Gibco, 31985–062). At the time of transfection, the medium was replaced with growth medium without FBS, and the transfection mix was prepared according to the manufacturer’s protocol. Cells where then incubated in normoxic or hypoxic conditions. 48 h later, EVs isolation and RNA extraction from EVs and cells was performed.

### EVs isolation

EVs were isolated from conditioned media (CM) by ultrafiltration using 100-kDa MWCO Spin-X 20 ml Concentrator (Corning) following the manufacturer’s instructions. To generate CM, 4 × 10^6^ cells were cultured in complete growth medium for 48 h (80% confluency) and maintained in normoxic (20% O_2_) or hypoxic (1.5% O_2_) condition. Media was then replaced with DMEM without FBS supplemented with 2 mM glutamine, 100 U/ml penicillin/streptomycin (Life Technologies) and 1 × MEM non-Essential Amino Acids (Biowest) for 48 h. CM was harvested, centrifuged to remove cellular debris (2000 g for 5 min), and filtered by 0.22 mm syringe filters (Millipore). 20 ml of CM were then loaded into the concentrator tube and centrifuged at 3000 g for 30 min at room temperature. This last step was repeated by adding 13 ml of PBS and centrifuged at 3000 g for 15 min at room temperature. EVs were collected and either used fresh or stored at − 80 °C. Similarly, 20 ml of cell culture media without FBS was ultrafiltrated, collected and stored at – 80 °C as negative control deprived of EVs (CTR). Total amount of proteins in EVs was quantified by Pierce BCA Protein Assays (ThermoFisher). The same procedure was performed with NB cells transfected with miRNA mimic, miRNA inhibitor and negative control.

### Quantification of EVs

EVs concentration was analyzed using a qNano system (Izon Science Ltd.) and Nanoparticle Tracking Analysis (NTA) with Nanosight NS300 (Malvern), according to the manufacturer's instructions. For qNano measurement, EVs were diluted 1:100 and measured using a NP100 nanopore (analysis range 50–330 nm) with 10 mbar pressure. Voltage and stretch were set to give a stable current between 100 and 120 nA. Data were analyzed using the Izon Control Suite software. For NTA measurement, samples were diluted 1:100 in PBS and analyzed under constant flow conditions at 25 °C. For each measurement, three 60 s videos were captured with a camera level of 14. Data were analyzed using NTA software with a detection threshold of 5.

### Transmission electron microscopy (TEM)

For electron microscopy analyses, one drop of purified EVs (~ 25 µl) was deposited on 400 mesh holey film grid. Filter paper (Whatman) was used to remove the excess liquid. EVs were then stained with 1% uranyl acetate for 2 min. For immunogold staining, EVs were incubated with mouse anti-CD81 (1:50; ab59477, abcam) for 30 min followed by secondary anti-mouse 10 nm protein A-gold conjugates (Sigma-Aldrich) for 30 min. Samples were observed with a Tecnai G2 (FEI) transmission electron microscope operating at 100 kV equipped with a Veleta CCD camera (Olympus Soft Imaging System) at the BioImaging Facility (Department of Biology, University of Padova).

### Bead-based multiplex assay

EVs surface protein profiling was performed using the MACSPlex Exosome kit (130–108-813, Miltenyi Biotec) which allows the detection of 39 surface epitopes (CD3, CD4, CD19, CD8, HLA DR, CD56, CD105, CD2, CD1c, CD25, CD49e, ROR1, CD209, CD9, SSEA4, HLA ABC, CD63, CD40, CD62p, CD11c, CD81, MCSP, CD146, CD41b, CD24, CD86, CD44, CD326, CD133, CD29, CD69, CD142, CD45, CD31, REA control, CD20, CD14, IgG1 control, CD42a). The samples were analyzed with a Cytoflex flow cytometer (Beckman Coulter) with at least 10,000 recorded events each. The calibration and gating were performed accordingly to manufacturer’s recommendations using the MACSPlex Exosome Setup Beads. Data were analyzed with FlowJo software and the median fluorescence values were background corrected. Gene Ontology (GO) analysis of upregulated proteins was performed with the online tool Search Tool for the Retrieval of Interacting Genes/Proteins (STRING).

### Western blotting

Total cell lysates were prepared using lysis buffer (Biosource International) supplemented with Phosphatase Inhibitor Cocktail 2 (P5726, Sigma Aldrich), Phosphatase Inhibitor Cocktail 3 (P044, Sigma Aldrich), Protease Inhibitor Cocktail (Sigma-Aldrich) and PMSF serine protease inhibitor (Sigma-Aldrich), and 10 µg of proteins were separated by SDS-PAGE using Bolt 4–12% Bis–Tris Plus gels (Invitrogen). Gels were transferred onto nitrocellulose membranes using Power Blotter Select Transfer Stacks and the Power Blotter System (Invitrogen). Membranes were blocked with I-Block reagent (ThermoFisher) and incubated with the following primary antibodies overnight at 4 °C: mouse monoclonal anti-calnexin (sc-23954, Santa Cruz); mouse monoclonal anti-CD63 (ab213090, abcam); mouse monoclonal anti-CD81 (ab59477, abcam); mouse monoclonal anti-GAPDH (GTX627408); mouse monoclonal anti-Cytochrome C (SC-13156); mouse monoclonal anti-HSP70/HSC70 (W27, SC-24). Membranes were then incubated with the appropriate peroxidase-conjugated secondary antibody, Goat anti-Mouse (G-21040). Images were acquired using Westar Hypernova ECL substrate (Cyanagen) and iBright Western Blot Imaging Systems (Invitrogen).

### Cell colony-formation assay

NB cells were seeded in 12-well plates (2 × 10^3^ cells/well) using MethoCult semi-solid medium (Stemcell Technologies) and incubated in the presence of 20 µg/ml of EVs for 15 days. Colony formation was analyzed by staining cells with MTT (0.5 mg/ml). Images were acquired using an EVOS FL Cell Imaging System (Thermo Fisher Scientific) and analyzed using ImageJ software (National Institutes of Health, MD, United States).

### Wound healing assay

NB cells (3 × 10^4^ cells/well) were seeded within each of the two cell culture reservoirs separated by a silicone wall (IBIDI). After 24 h, the silicone insert was removed, and cells were incubated with fresh medium containing 20 µg/ml of EVs or control. Images were acquired using an EVOS FL Cell Imaging System (ThermoFisher) and cell migration analysis was performed using ImageJ.

### Transwell assay

Cell invasion was evaluated using 24-well transwell inserts (pore size: 8 μm, Corning, 353097) coated with 50 μl of Matrigel (Corning, 354234; 13.1 mg/ml). A total of 4 × 10^4^ cells suspended in 200 μl of serum free medium and EVs (20 μg/ml) was plated on top of the Matrigel-coated membranes while 500 μl of complete growth medium was added in the lower chamber. After 48 h, cells inside transwell inserts were removed by gently wiping with a cotton swab. The cells that invaded the lower surface of the membrane were stained with 0.1% crystal violet solution. Pictures of crystal violet-stained cells adhering to the lower surface of the transwell membranes were taken with Primovert microscopy (Zeiss) and the number of invaded cells was counted using ImageJ.

### EVs membrane labeling and imaging

EVs were labeled with the fluorescent Vybrant DiO Cell-Labeling Solution (V22886, ThermoFisher) as recommended by the manufacturer. Briefly, EVs were resuspended in 1 ml of PBS 1X mixed with 5 μl of DiO cell-labeling solution for 30 min at 37 °C. Labeled EVs were washed with 13 ml of PBS 1X, then loaded into the concentrator tube and centrifuged at 3000 g for 15 min at room temperature to remove free DiO dye. The same protocol was performed for negative controls. Cells were plated in 4-well Chamber Slides. After reaching the appropriate density, 20 µg/ml of labeled EVs or control were added (time zero) and incubated for 8, 24, and 48 h. Cells were then fixed in 3.7% formaldehyde (Sigma-Aldrich), permeabilized with 0.1% Triton X-100 (Sigma-Aldrich) and stained with 50 µg/ml of fluorescent phalloidin-TRITC (P1951, Sigma-Aldrich) and DAPI (10236276001, Roche). Imaging was performed on a Zeiss Axio Imager M1 epifluorescence microscope (Zeiss, Oberkochen, Germany). Fluorescence intensity was measured using ImageJ.

### miRNA assay and analysis

miRNAs detection and quantification were performed using the FirePlex® miRNA assay (abcam, Cambridge, MA, USA), a technology not requiring RNA purification nor pre-amplification, with multiplexing resulting from specific particle coding identifying individual miRNAs with picomolar sensitivity and high specificity. Data acquisition for miRNAs expression was performed by flow cytometry [15]; Arbitrary Units (AU) of fluorescence per miRNAs target are proportional to the average amount of fluorescent targets bound to the particles. Data were analyzed using the FirePlex™ Analysis Workbench software [16]. miRNAs with a fluorescence value over the detection threshold after subtraction of background have been considered for the analyses (AU > 4.71). Visualization plots were generated with custom R codes.

### Principal Components Analysis

Principal component analysis (PCA) is a mathematical technique for reducing the dimensionality of multivariate datasets, whilst minimizing information loss and avoiding distributional assumptions over the populations being analyzed [19]. Principal components (PCs) are uncorrelated linear combinations of the original variables and provide a synthetic description of the major trends in the data through maximization of the explained variance [20]. Finding the PC is equivalent to solving an eigenvalue/eigenvector problem on the covariance (or correlation) matrix computed from the data. PCA decomposes the data matrix into scores and loadings pair vectors that summarize the information on the correlations existing between samples and variables. PCA was conducted using the software PLS-Toolbox (Eigenvector Research, Inc.) within the MATLAB® computational environment (MathWorks, Natick).

### RNA extraction and quantitative PCR

NB cells were seeded in 6-well plates (5 × 10^5^ cells/well) and allowed to adhere for 24 h, then they were incubated with fresh medium containing 20 µg/ml of EVs for an additional 24 h. Total RNA from treated cells was extracted using Trizol reagent (15596026, Invitrogen), and real-time PCR was performed using Platinum SYBR Green (7900 Applied Biosystems) according to manufacturing instruction. Relative mRNA expression for each gene was analyzed by the ddCt method using *GUS* as housekeeping gene. A list of all primers is provided in Additional file 2**: **Table S1. EVs RNA from both non transfected and transfected SK-N-AS and SK-N-DZ cell culture supernatant cultured in all oxygenation conditions was extracted using exoRNeasy Maxi Kit (Qiagen, 77164). Before extraction, CM was harvested, centrifuged to remove cellular debris (500 rcf, 5 min), and filtered using 0.22 μm syringe filters (Sartorius, 16534) to exclude particles larger than 0.8 μm. Total RNA, including miRNA, was quantified using a Nanodrop spectrophotometer (Thermo Fisher Scientific). Isolated RNA was used to synthesize cDNA using the TaqMan Advanced miRNA cDNA Synthesis Kit (Applied Biosystem, A25576) according to manufacturer’s instruction. Real-Time PCR was set up using the TaqMan Fast Advanced Master Mix (Applied Biosystems, 4444963) and TaqMan Advanced miRNA Assays: the target miRNA hsa-miR210-3p (477970_mir) and hsa-miR-16-5p (477860_mir) as an endogenous control (both Applied Biosystem). The relative miRNA expression was analyzed by the ddCt method.

### Zebrafish and Xenotransplantation

The Casper strain of zebrafish (*Danio rerio*) and the transgenic zebrafish line Tg(*fli1:EGFP*) were maintained and handled in accordance with European Animal Welfare Legislation (Directive 2010/63/EU) in the ZebraLab facility at IRPCDS. Fertilized eggs were obtained from natural spawning. Embryos were kept at 28.5 °C in methylene blue-containing water until they reached the embryonic stage for injection. We prepared cells and performed xenotransplantations as described previously [21]. Briefly, SK-N-AS cells were seeded in 6-well plates (5 × 10^5^ cells/well) and allowed to adhere for 24 h, then they were incubated with fresh medium containing 20 µg/ml of EVs. After 48 h and prior to injection, SK-N-AS cells were labeled with Vybrant DiI Cell-Labeling Solution (Invitrogen, V22885) according to the manufacturer's instructions. Before injection, cells were resuspended in PBS 1X and kept on ice until transplantation. For transplantations, 48-h post fertilization (hpf) embryos were dechorionated, anesthetized with 200 mg/l Tricaine (Sigma-Aldrich) and placed on a Petri dish containing a solution of 2% agarose in fish water. Cell suspension was loaded into borosilicate glass capillary needles (1.0 mm in diameter, World Precision Instruments, Sarasota, FL, USA) and injected into the center of the yolk sac of Casper embryos or into the Duct of Cuvier of Tg (*fli1:EGFP*) embryos using a pneumatic microinjector (World Precision Instruments) under the SMZ1500 fluorescent stereomicroscope (Nikon). By controlling injection pressure and duration, the number of injected cells was set at approximately 100–150 cells per embryo as determined by standard cell counting of injection droplets. Injected embryos were transferred to fresh fish water for recovery during 1 h at 28.5 °C and then incubated at 34 °C for the rest of the experiment.

### Confocal Imaging and Analysis

Prior to formal analysis, 1 hpi (hour post injection) embryos were evaluated for red fluorescence of tumor cells in the injection site using a SMZ1500 fluorescent stereomicroscope (Nikon) to exclude those with tumor cells located outside of the injection site. We then evaluated tumor cells migration and invasion by confocal microscope (Zeiss LSM800 Airyscan) 24 hpi. To evaluate the migration ability of NB cells in Casper embryos, the whole embryo and the caudal plexus were imaged at 2.5X and 10X magnification, respectively. Tumor cells number localized in the caudal plexus region was automatically counted using ImageJ. To evaluate the invasion ability of NB cells in *Tg(fli1:EGFP),* embryos were imaged at 20X magnification (z-stack) to provide a single cell view, and invaded tumor cell were manually counted. The invasion rate was calculated as follows: invasion rate (%) = (the number of zebrafish with cell extravasated/the initial number of xenografted zebrafish) × 100. At least 15 embryos per conditions from at least 3 independent experiments were used for each test.

### Statistical analysis

Graphs and statistical analyses were performed using GraphPad Prism software. All data in graphs are from at least three independent experiments ± SEM. In the box-and-whiskers graphs, box margins are the 25th and 75th percentile, the center line is the median, and the whiskers the minimum and maximum values. Statistical significance was determined using unpaired Student’s t-test. Asterisks indicate a significant difference between the treated and the control group, unless otherwise specified. * p < 0.05, ** p < 0.01, *** p < 0.001, **** p < 0.0001.

## Results

### Characterization of EVs from NB cells cultured at different oxygen tensions

**Fig. 1:**
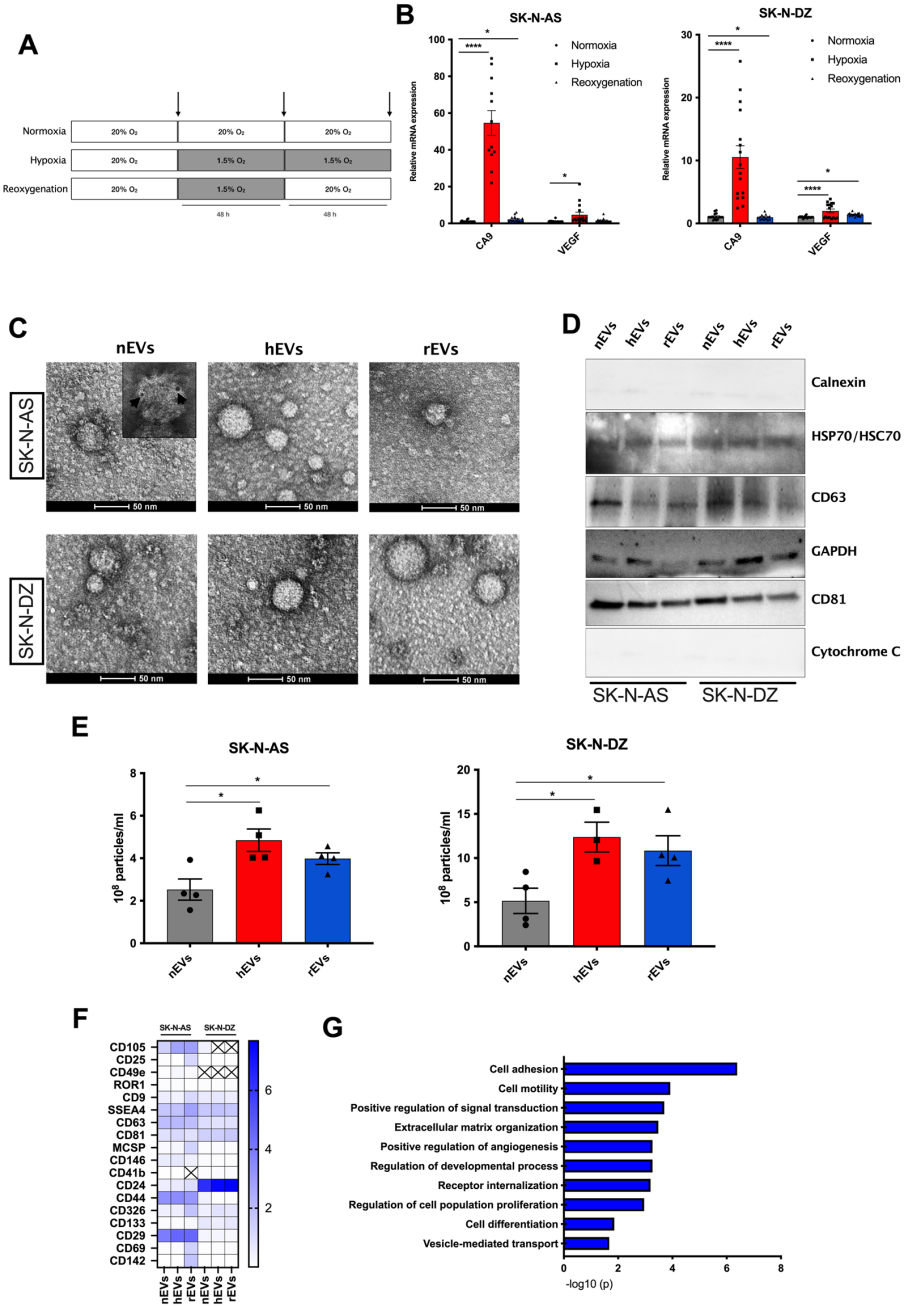
Isolation and characterization of EVs from NB cell culture conditioned media. **A**. Schematic representation of the experimental flow. Arrows indicate media changes and collection of CM for EVs isolation. **B**. Relative mRNA expression of the hypoxia markers CA9 and VEGF measured by qPCR in SK-N-AS and SK-N-DZ cells cultured in normoxia (grey box), hypoxia (red box), and reoxygenation (blue box) conditions. *p < 0.05 and ****p < 0.0001 vs normoxia. **C**. Representative TEM images show the morphology of EVs. Insert indicates immunogold labeled EVs with anti CD81 antibody. **D**. Validation of EVs marker expression by western blot analysis. **E**. EVs concentration normalized by number of SK-N-AS and SK-N-DZ cells cultured in normoxia (grey box), hypoxia (red box), and reoxygenation (blue box) conditions as quantified by qNano. *p < 0.05 vs normoxia **F.** Heatmap depicting the background-corrected signals of 18 out the 39 markers expressed on the surface of NB-EVs. **G**. Bar graph showing enrichment of the top10 biological signaling pathway components of the upregulated proteins in common between the two cell lines

To address the structural and functional features of EVs isolated from NB cells exposed to different oxygen tensions, we chose 2 widely used NB cell lines that differ for their *MYCN* status: non-*MYCN* amplified SK-N-AS and *MYCN*-amplified SK-N-DZ line. NB cells were cultured for 2 days at 20% O_2_ (normoxia), or 1.5% O_2_ (hypoxia and reoxygenation conditions); media was then replaced with DMEM without FBS, and cells were cultured for 2 additional days at 20% O_2_ (normoxia and reoxygenation conditions), or at 1.5% O_2_ (hypoxia). CM was collected and used for EVs isolation (Fig. 1A). We verified activation of the hypoxia pathway in NB cells by quantitative PCR (qPCR) of two key hypoxia markers: Carbonic anhydrase 9 (*CA9*) and vascular endothelial growth factor (*VEGF*), both transcriptionally activated by HIFs [22, 23]. The expression of *CA9* and *VEGF* was upregulated in hypoxic conditions in both cell lines. Data for SK-N-DZ cells suggest that transient hypoxia might be sufficient to induce a memory-retaining signature even following reoxygenation (Fig. 1B).

We next analyzed the biophysical and morphological features of isolated EVs from hypoxic (hEVs), reoxygenation (rEVs), and normoxic (nEVs) conditions. Morphological analysis by transmission electron microscopy (TEM) revealed a homogeneous population of vesicles with a size of ~ 40 nm (Additional file 1: Figure S1A), unaffected by the oxygen tension at which NB cells were cultured (Fig. 1C). We also performed immunogold labelling EM using anti-CD81 to further confirm the identity of isolated EVs (insert in Fig. 1C). Confirming published evidence [24], NanoSight and qNANO consistently measured larger particle diameter (~ 100 nm) than TEM (Additional file 1: Figure S1B, C). We used immunoblot to verify the expression of EVs markers including CD63, CD81, HSP70/HSC70 and GAPDH, and the absence of negative control Calnexin and Cytochrome C, also confirming the absence of cell contaminates in the EVs preparations (Fig. 1D). When EVs concentrations were normalized to cell number at the time of harvest, SK-N-DZ cells secreted significantly more EVs compared to SK-N-AS in all tested conditions, in agreement with published evidence that MYCN amplified cells produce more vesicles compared to MYCN not-amplified cells [25]. Moreover, EVs secretion from both cell lines was higher for hEVs and rEVs compared with nEVs. We measured: 2- and 2.4-fold for hEVs, and 1.5- and 2.1-fold increase for rEVs from SK-N-AS and SK-N-DZ cells, respectively (Fig. 1E). These data indicate that hypoxic as well as reoxygenation NB cultures release more EVs that are comparable in size and shape with those released from NB cells cultured in normoxic conditions.

### EVs surface protein profiles are not affected by oxygenation conditions

Membrane surface proteins of vesicles are important in intercellular communication, are an attractive source of disease markers, and are mediators of specific bio-distribution and uptake by receiving cells [26, 27]. We profiled the surface protein composition of NB EVs using the MACSplex technology. EVs isolated from SK-N-AS and SK-N-DZ cells cultured in the three oxygenation conditions were incubated with 39 capture antibody beads (37 surface epitopes and 2 isotype controls) and stained with a cocktail of CD9-, CD63-, and CD81-APC antibodies. NB derived EVs were positive for 18 out of the 37 tested surface markers (CD105, CD25, CD49e, ROR1, CD9, SSEA4, CD63, CD81, MCSP, CD146, CD41b, CD24, CD44, CD326, CD133, CD29, CD69, CD142). We confirmed that EVs markers such as the tetraspanins CD9, CD63 and CD81 were enriched on NB-derived EVs (Additional file 1: Figure S1D, E). A heatmap of surface markers relative abundance showed that cellular origin or culture conditions did not affect them, except for CD105 and CD49e that were selectively expressed on EVs from the SK-N-AS line (Fig. 1F). Overall, hypoxic or normoxic EVs did not differ for their surface markers. A gene ontology (GO) enrichment analysis on the shared markers expressed in both cell lines at the different oxygenation conditions, indicated that NB EVs display signatures linked to cancer aggressiveness, with enrichment of the following GO term: cell adhesion (10 proteins, p = 0.0000004, GO:0,007,155), cell motility (8 proteins, p = 0.0001, GO:0048870), proliferation (8 proteins, p = 0.001, GO:0042127), vesicle-mediated transport (6 proteins, p = 0.02, GO:0016192), extracellular matrix organization (5 proteins, p = 0.0003, GO:0030198) and angiogenesis (4 proteins, p = 0.0005, GO:0045766) (Fig. 1G**).** Taken together, these data indicate that the oxygen tension at which NB cells are cultured does not affect the surface markers of the shed EVs and that the surface of NB EVs is enriched with proteins involved in multiple features of cancer aggressiveness.

### hEVs promote migration and invasion of target cells

**Fig. 2:**
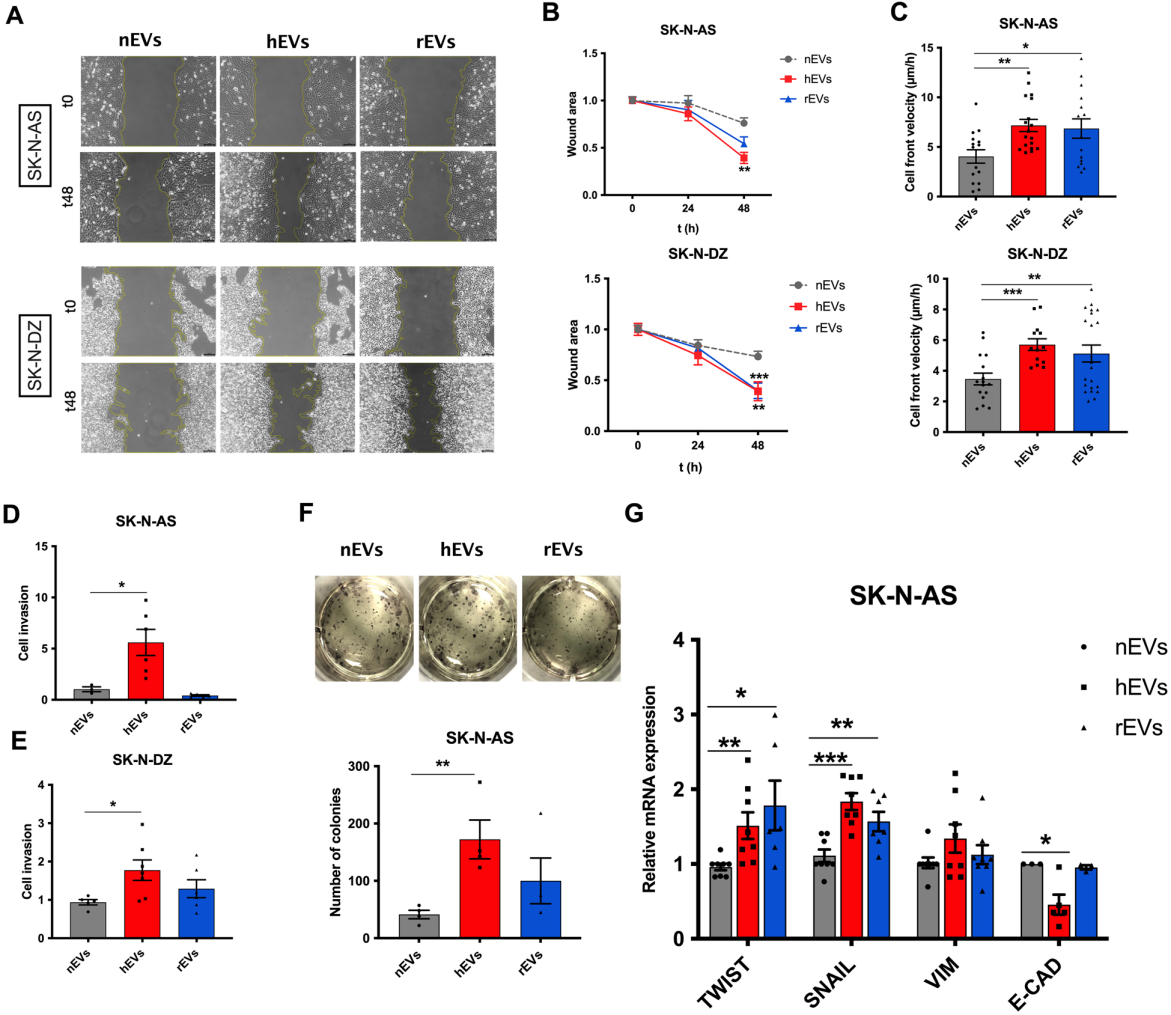
hEVs affect migration and invasion ability of NB cells. All experiments are performed on SK-N-AS and SK-N-DZ cells treated with EVs from SK-N-AS and SK-N-DZ cells cultured in normoxic, hypoxic and reoxygenation conditions. **A**. Representative images of wound healing assay; images were acquired at time zero (t0), 24 (not shown) and 48 h after treatment. Scale bar, 100 µm. **B**. Wound closure areas and **C**. cell velocity (μm/h). *p < 0.05 and ***p < 0.001 vs normoxia. **D**, **E**. Quantification of cells migrated toward the Matrigel layer (average of 5 picture fields at 10 × magnification). All values are mean ± SEM from at least 3 independent experiments. **F.** Representative images of colony forming assay and quantitative. Values are expressed as median ± SEM from at least 3 independent experiments. *p < 0.01 and **p < 0.01 vs normoxia. **G.** Relative mRNA expression of the genes involved in EMT and invasiveness (TWIST, SNAIL1, VIMENTIN and CDH1) were measured by qPCR in SK-N-AS cells after treatment with EVs from SK-N-AS cells cultured in normoxia (grey box), hypoxia (red box), and reoxygenation (blue box) conditions. Values from at least n = 3 independent experiments. *p < 0.05, **p < 0.01 and ***p < 0.001 vs normoxia. All quantifications were performed with Image J

Given that EVs surface includes several proteins promoting adhesion and motility, we investigated whether these EVs affect the migration and invasiveness of naïve NB cells. First, we evaluated the internalization of EVs in target SK-N-AS cells by fluorescence microscopy. EVs isolated from SK-N-AS cells cultured in normoxic, hypoxic and reoxygenation conditions were stained with the DiO labeling solution and added to fresh SK-N-AS cells culture medium for 8 to 24 h. Internalization of fluorescently labeled EVs (green) inside the phalloidin-stained cytoplasm of SK-N-AS cells (red) started after 8 h and increased over time, and we measured an increased uptake for hEVs and rEVs compared to their normoxic counterparts (Additional file 1: Figure S2A and S2B). We then investigated if EVs affected two key parameters linked to tumor aggressiveness: migration and invasiveness. In a classic wound healing assay, treatment for 48 h with hEVs and rEVs increased the migration ability of SK-N-AS and SK-N-DZ cells, as cells closed larger wound areas than those treated with nEVs (Fig. 2A, B). Calculations of cells velocity (µm/h) in all experimental conditions using measures of the wound width at t = 0 and t = 48 h proved that cells treated with hEVs moved faster than cells treated with nEVs **(**Fig. 2C**)**; a similar trend was measured in cells treated with rEVs compared to nEVs.

To determine EVs role on the invasion ability of SK-N-AS and SK-N-DZ cells, we performed a transwell assay proving that hEVs enhanced the invasiveness of SK-N-AS **(**Fig. 2D**)** and SK-N-DZ **(**Fig. 2E**)** cells compared to nEVs-treated cells, whereas no significative differences emerged between rEVs and nEVs-treated cells. With a colony formation assays in semisolid medium adding EVs to cells and counting colonies after 15 days, we then verified that hEVs modified the clonogenic ability of SK-N-AS cells increasing the number and size of colonies compared to nEVs-treated cells (Fig. 2F). These results suggest that hypoxia induces production of EVs able to promote motility and invasiveness of naïve NB cells, a behavior partially conserved even after oxygen is restored, supporting the hypothesis that cells retain a memory of the exposure to the hypoxic environment.

Finally, quantitative PCR (qPCR) analysis of genes involved in epithelial-mesenchymal transition (EMT) and invasiveness resulted in increased expression of *TWIST* and *SNAIL* and a loss of *E-CADHERIN* in cells treated with hEVs compared with nEVs **(**Fig. 2G**)**.

### EVs “miRNome” cargo analysis at different oxygen tension

**Fig. 3:**
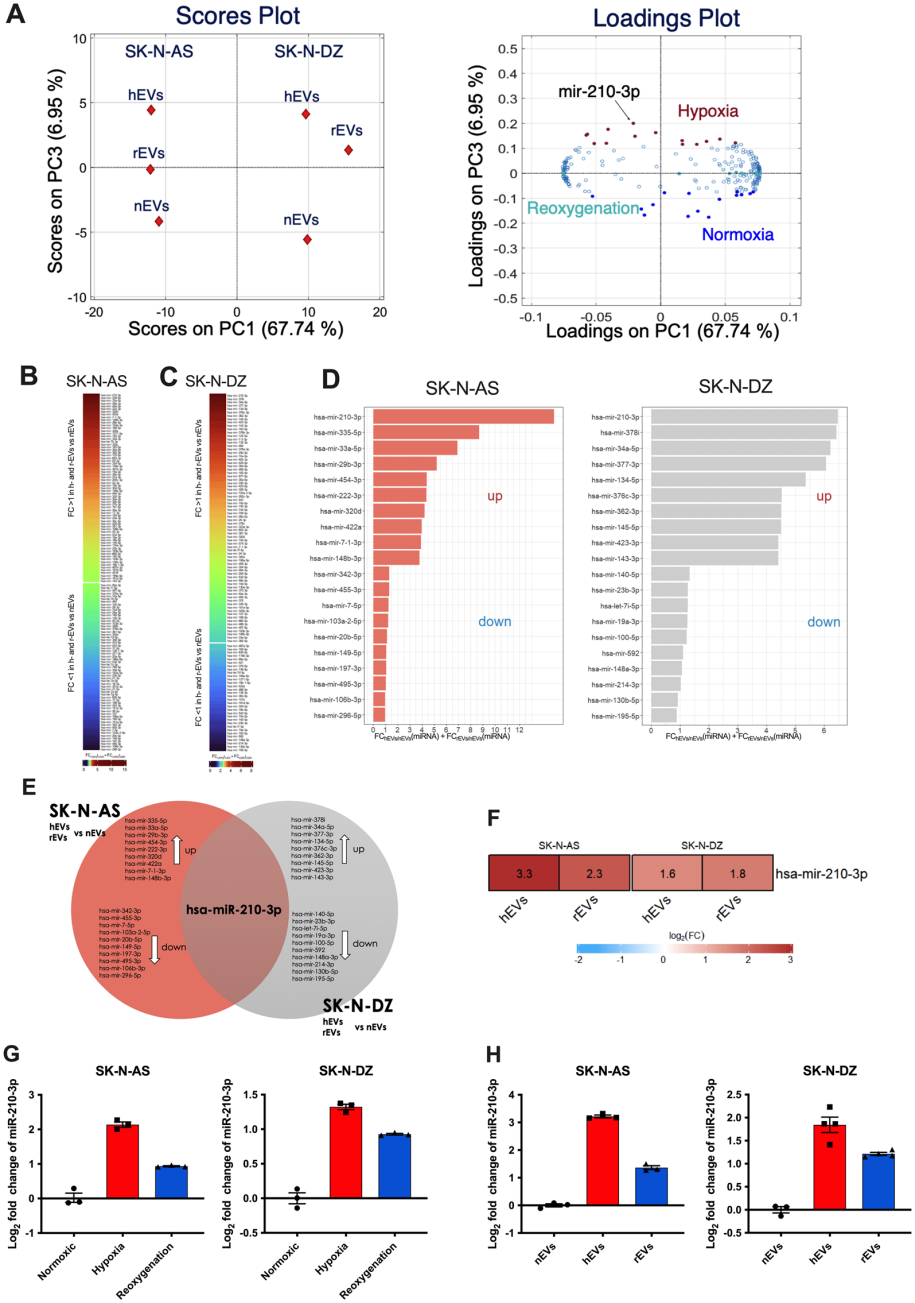
miR-210-3p is highly expressed in hypoxic cells and their derived EVs. **A**. Scores and loading plots of the PCA model trained on the miRNA dataset (n = 6). The plot displays the first and third principal components. Color key on the loadings plot: (red) miRNAs highly expressed in hEVs; (light blue) miRNAs highly expressed in rEVs; (dark blue) miRNAs highly expressed in nEVs. **B.** Heatmap of the 130 miRNAs derived from SK-N-AS EVs. **C.** Heatmap of the 160 miRNAs derived from SK-N-DZ EVs. **D.** The histograms show two sets of the 10 most upregulated and 10 most downregulated miRNAs modulated in hypoxia and reoxygenation in both SK-N-AS and SK-N-DZ. **E.** Venn diagram highlights miR-210-3p as the common miRNA modulated in hypoxia and reoxygenation. **F.** Heatmap shows the fold change of miR-210-3p in normoxia and reoxygenation condition. **G, H.** qPCR analysis of miR-210-3p levels in SK-N-AS and SK-N-DZ cells (**G**) and EVs (**H**) cultured in normoxic, hypoxic and reoxygenation conditions. Data are represented as log2(2-ΔΔCt), miR-16-5p was used as housekeeping gene. Values are expressed as mean ± SEM

To understand the mechanisms of EV signaling, we profiled the miRNome cargo of EVs produced by NB cell lines cultured in normoxic, hypoxic and reoxygenation condition using FirePlex^®^ miRNA assay (Qixin Leng et al. 2018), analyzing a total pool of 405 miRNAs (Additional file 3: Table S2). Retaining miRNAs with fluorescence intensity above the detection threshold (AU > 4.71) in each condition resulted in a panel of 250 miRNAs for further analysis (Additional file 1: Figure S3). We first employed principal component analysis (PCA) on the standardized miRNAs expression matrix to assess if miRNAs exhibit different expression clusters patterns between cell lines and among the three oxygenation statuses. A model with three principal components (PCs) captures 90.3% of the original variability, with the following PC proportions: 67.74% (PC1), 15.60% (PC2) and 6.95% (PC3). Noticeably, the main difference between EVs is ascribable to the cell line from which they originated (Fig. 3A). Discrimination among the hypoxia, reoxygenation and normoxia statuses is provided by PC3. The miRNAs characterizing the three oxygenation conditions were chosen as the 7% with the highest loading on PC3 (hypoxia), around zero (reoxygenation) and lowest loading for the normoxia conditions (highest in absolute value, but negative in sign). Our findings confirm the molecular heterogeneity of NB cell lines, which mirrors the different clinical appearance of HR NB patients.

To prioritize hypoxia-associated miRNAs, we implemented the workflow described in Additional file 1: Figure S4 to identify putative key miRNAs for subsequent validation. Such miRNAs should be modulated, either up- or down-, in hypoxia and reoxygenation in both cell lines. As such, we charted two lists of *i*. 130 and *ii.* 106 EVs miRNAs from SK-N-AS and SK-N-DZ cells, respectively. List *i*. comprises miRNAs that exhibit the same trend towards up- or downregulation when comparing SK-N-AS derived hEVs and rEVs vs nEVs (Fig. 3B). A fold-change > 1 suggests a tendency towards upregulation (< 1 towards downregulation) of each miRNA in the SK-N-AS cell line in hEVs and rEVs with respect to their normoxic counterpart. List *ii*. was obtained adopting the same rationale to EVs derived from SK-N-DZ cells (Fig. 3C). miRNAs in the two lists were then ranked considering the sum of their fold-change. We subset the 10 most upregulated and 10 most downregulated from each list, obtaining two sets of 20 miRNAs each (Fig. 3D). Their intersection revealed miR-210-3p as the common, modulated in hypoxia and reoxygenation in both cell lines (Fig. 3E, F). Evaluation of miR-210-3p expression resulted in a significantly higher expression in hypoxic compared to normoxic conditions, both in SK-N-AS and SK-N-DZ cell lines (Fig. 3G) and in their derived EVs (Fig. 3H); a behavior that was also conserved in reoxygenation. These results confirmed that miR-210-3p was highly expressed by cells and EVs cultured in hypoxic conditions; the relative expression in cells cultured in reoxygenation conditions and their derived EVs was also higher than normoxic cells and EVs, supporting the hypothesis that cells retain a memory of the pre-exposure to hypoxic environments.

### miR-210-3p levels in EVs define migration and invasiveness of target cells

**Fig. 4:**
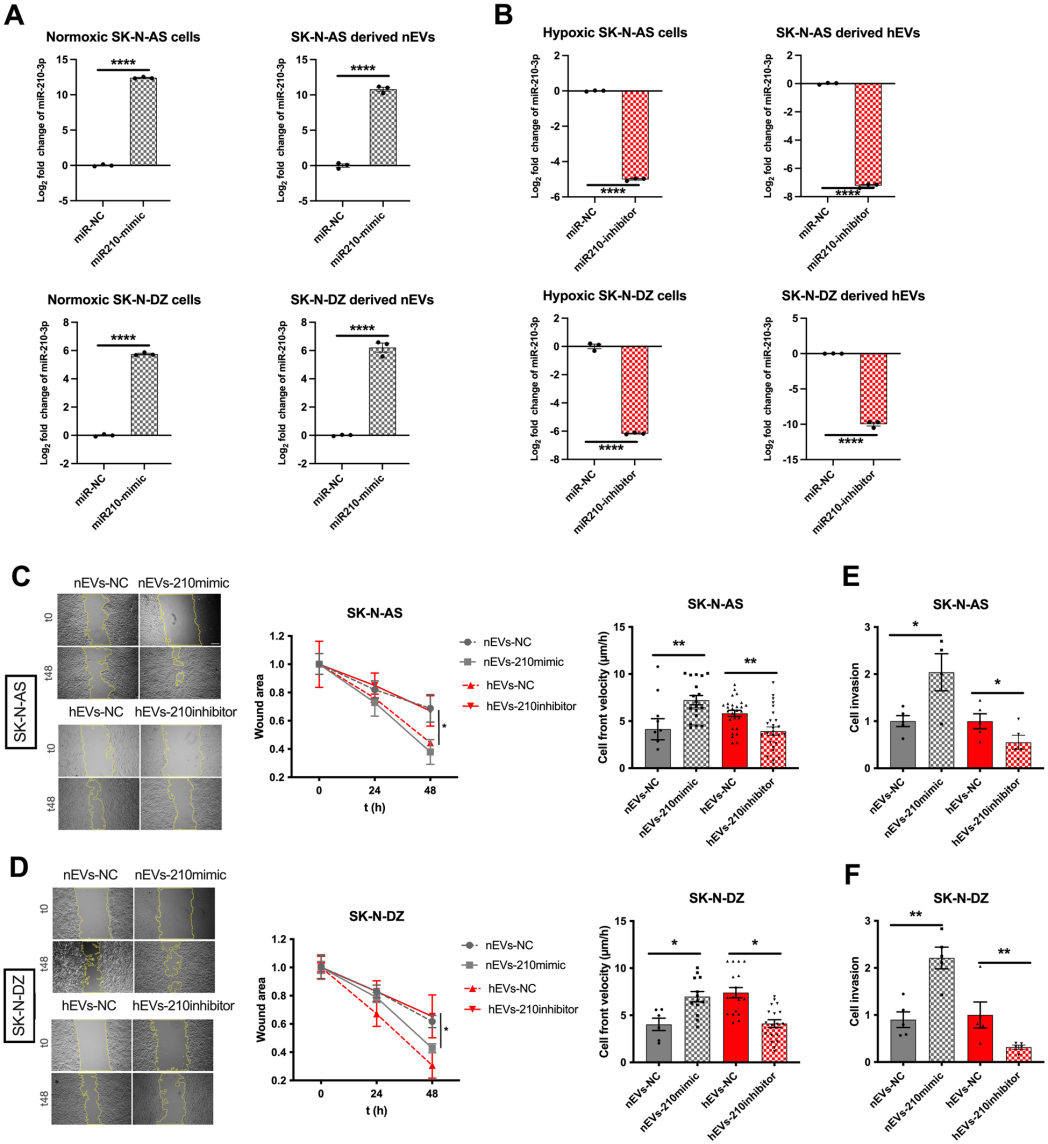
EVs derived from transfected cells affect migration and invasiveness of NB cells. **A**. qPCR analysis of miR-210-3p levels in SK-N-AS and SK-N-DZ cells and derived EVs transfected with miR-210-3p mimic and cultured in normoxic condition. **B**. qPCR analysis of miR-210-3p levels in NB cells and EVs transfected with miR-210-3p inhibitor and cultured in hypoxic condition. Data are represented as log2(2-ΔΔCt). Values are expressed as mean ± SEM. ****p < 0.0001 vs miR-NC. **C-D.** Representative images of wound healing assay performed on cells treated with EVs from transfected NB cells with miR-210-3p mimic and miR-210-3p inhibitor; images were acquired at the time of EVs addition (t0) and 48 h after treatment (t48). Magnification 10X. Scale bar, 100 µm. Wound closure areas and cell velocity (µm/h) were quantified with Image J. Values are expressed as mean ± SEM from 3 independent experiments. **E–F.** Quantification of cells invaded toward the Matrigel layer (average of 5 picture fields at 10 × magnification) via Image J. nEVs-210mimic data are normalized on the averaged value for nEVs, hEVs-210inhibitor are similarly normalized on hEVs-NC. Values are mean ± SEM

Given miR-210-3p levels are modulated by oxygenation statuses, we postulated that it could represent a key cargo mediating the biological effects of hEVs. To verify this hypothesis, we genetically regulated its levels in SK-N-AS and SK-N-DZ cells cultured in normoxic, hypoxic or reoxygenation conditions and measured if miR-210-3p levels alone could influence the ability of EVs to promote migration and invasion of target cells. Given our previous findings, we focused on increasing miR-210-3p levels in nEVs and lowering it in hEVs. While transfection with miRNA210 mimics or inhibitors did not alter EVs morphology **(**Additional file 1: Figure S5A)**,** markers (Additional file 1: Figure S5B) and size (Additional file 1: Figure S5C), they modulated miR-210-3p expression compared to cells transfected with miRNA-NC in both SK-N-AS and SK-N-DZ cells and in their derived EVs **(**Fig. 4A, B**).** Transient transfection with miR210-mimic was successful in inducing increased miR-210-3p expression in normoxic cells and their derived EVs, while transfection with miR210-inhibitor decreased its expression in hypoxic cells and their derived EVs. Target cells were cultured in normoxic conditions, ensuring maintenance of basal expression levels of miR-210-3p. Treatment of cells cultured in normoxic conditions with EVs from miR210-mimic transfected NB cells, stimulated their migration and velocity **(**Fig. 4C, D**),** independently on the oxygenation condition of the transfected EVs-releasing NB cells (Additional file 1: Figure S6A). Furthermore, miR-210-3p overexpressing nEVs also stimulated invasion (measured in a classic transwell assay, Fig. 4E, F), independently on the oxygenation condition of the parental releasing cells (Additional file 1: Figure S6B). These findings indicate that EVs overexpressing miR-210-3p derived from normoxic cells stimulate migration and invasion of target NB cells like hEVs. Next, we silenced miR-210-3p in cells cultured in hypoxic conditions and verified whether EVs derived from these cells retained the ability to promote migration and invasion of target NB cells. As hypothesized, these EVs were unable to stimulate migration (Fig. 4C, D**)** and invasion (Fig. 4E, F) as efficiently as EVs derived from control transfected cells, despite the hypoxic culture conditions. Again, this behavior was conserved in cells treated with EVs isolated from NB cells transfected with miR-210-3p inhibitor and cultured in normoxia, hypoxia and reoxygenation conditions (Additional file 1: Figure S6C, D).

Taken together, these results establish a role for miR-210-3p as a key mediator of the biological effects of hypoxic EVs in target cells.

### miR-210-3p levels in EVs influence xenotransplanted tumor cells migration in zebrafish embryos

**Fig. 5:**
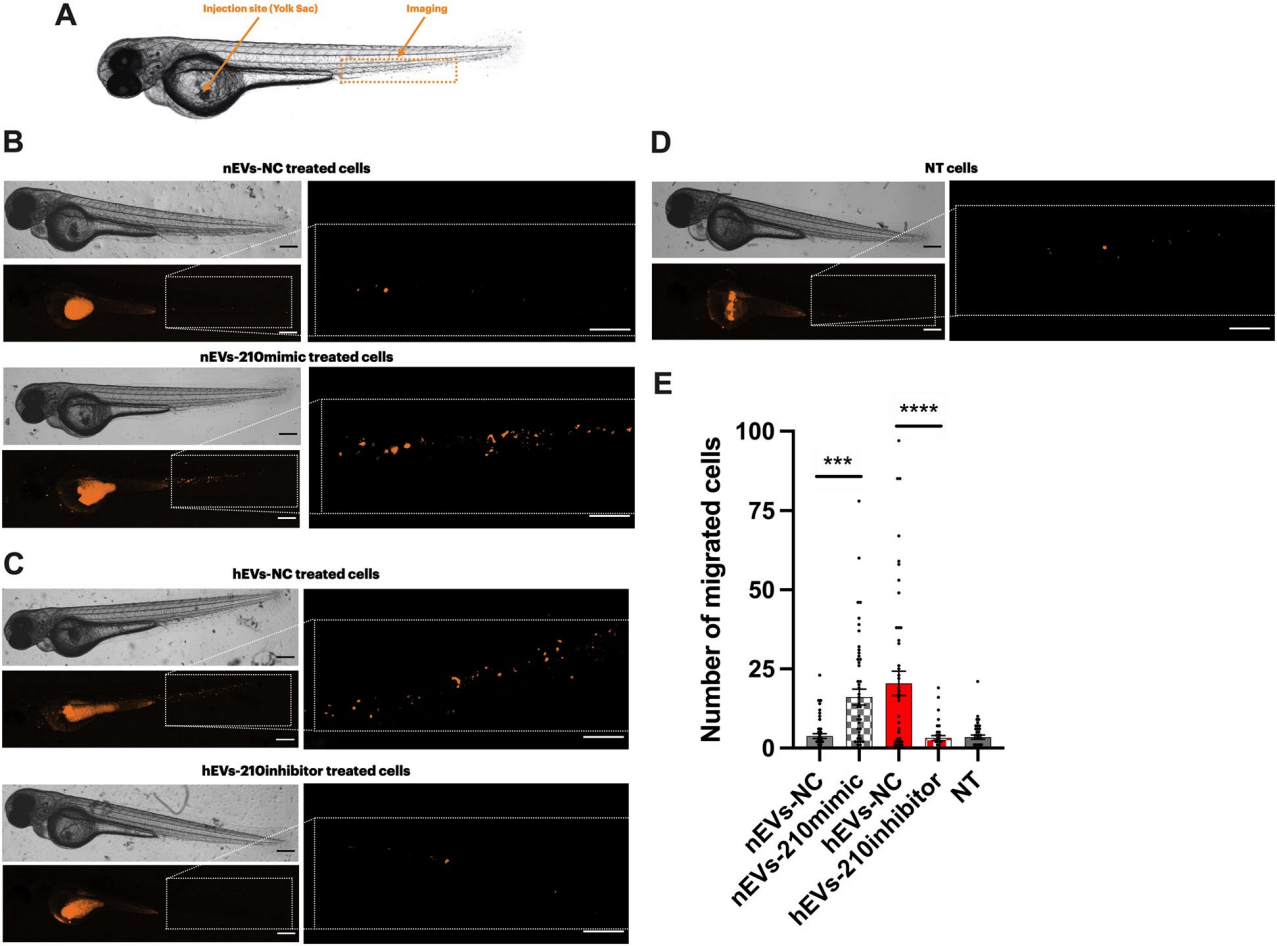
miR-210-3p levels in EVs influence xenotransplanted tumor cells migration in zebrafish embryos. SK-N-AS cells were labelled with DiI dye and injected into the Yolk sac of 48 hpf Casper strain zebrafish (n = 45 per condition). Migration ability was quantified by the number of cells migrated from the site of injection to the caudal plexus. All images show xenografts anterior to the left, posterior to the right, dorsal up and ventral down. **A.** Schematic representation of the site of injection (yolk sac) and the site of confocal acquisition in the caudal plexus region. **B**, **C**, **D.** Representative confocal images (2.5X magnification) of zebrafish embryo xenografts at 24 hpi showing cells injected in the yolk sac and cells migrated to the caudal plexus region. Scale bar 200 µm. Representative confocal images (10X magnification) of migrated cells in the caudal plexus region at 24 hpi. Scale bar 100 µm **E.** Quantifications of migrated cells in the tail region at 24 hpi. Values are mean ± SEM

To determine whether the level of miR-210-3p in NB EVs affects cells migration in zebrafish xenograft model, we injected approximately 100–150 SK-N-AS cells treated with EVs into the yolk sac of each Casper zebrafish embryo at 48 h post fertilization (hpf) (Fig. 5A) [28] and assessed the ability of tumor cells to enter circulation and migrate from the injection site to the caudal plexus region. A quantification of the tumor cells in the plexus region 24 h post injection (hpi) revealed that cells treated with nEVs-210mimic migrated 3.3 times more compared to those treated with nEVs-NC (Fig. 5B, E). Moreover, embryos injected with hEVs-210inhibitor treated cells had 5.4 times less migrated cells from the yolk sac to the plexus region compared to embryos injected with hEVs-NC treated cells (Fig. 5C, E). Finally, counts for embryos injected with cells not treated with EVs did not show any significant difference from the negative controls (Fig. 5D, E).

These data indicate that cells pre-treated with EVs isolated from cells transfected with miR-210-3p-mimic and cultured in normoxic condition have a higher migration ability in vivo compared to cells pre-treated with EVs isolated from cells transfected with miR-210-3p-NC. Furthermore, cells pre-treated with EVs isolated from cells transfected with miR-210-3p-inhibitor and cultured in hypoxic condition were apparently unable to migrate in vivo as efficiently as normoxic cells pre-treated with hEVs. These findings provide evidence that the expression level of miR-210-3p might influence the behavior of cells in an in vivo xenograft model, inducing a more aggressive phenotype.

### miR-210-3p levels in EVs induce tumor cells extravasation and caudal tail invasion in zebrafish xenograft embryos

**Fig. 6:**
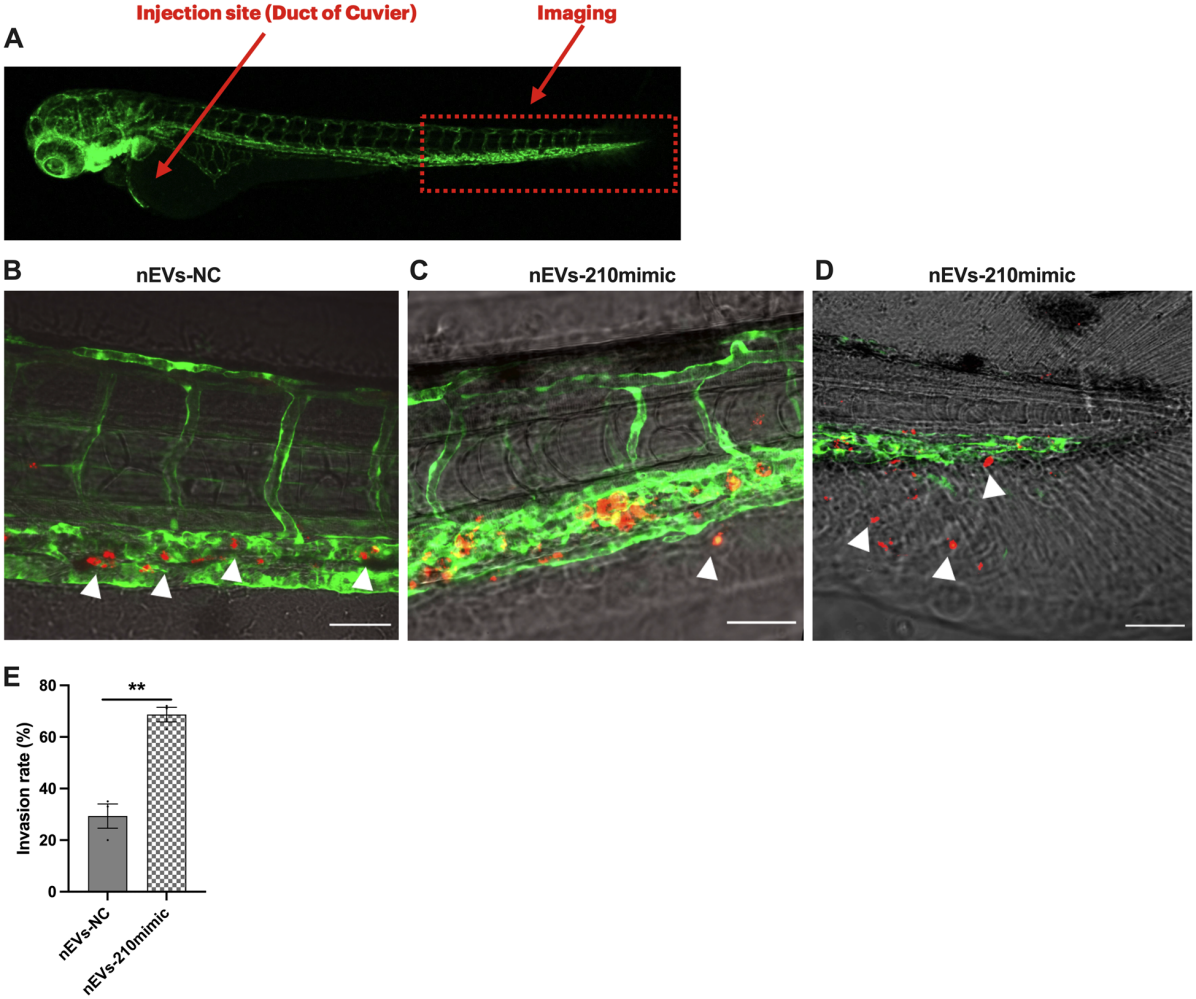
Extravasation and invasion of NB cells treated with nEVs-210mimic in zebrafish embryos. SK-N-AS cells were labelled with DiI dye and injected into the Duct of Cuvier of 2 dpf Tg(fli1:EGFP) zebrafish embryo (n = 45 per condition). All images show xenografts anterior to the left, posterior to the right, dorsal up and ventral down. **A**. Schematic representation of the site of injection (Duct of Cuvier) and the site of z-stack confocal acquisition in the caudal plexus region. **B**. Representative z-stack images of NB cancer cells (in red, white arrows) inside the vasculature (in green) that did not extravasate or invade the avascular tail region. Scale bar 50 µm. **C.** Representative z-stack images of NB cancer cells (in red, white arrows) extravasated through the vasculature (in green). Scale bar 50 µm. **D**. NB cells (in red, white arrows) invaded the avascular tail region (vasculature in green). Scale bar 50 µm. **E**. Quantification at 24 hpi revealed a higher number of embryos with invaded cells in those injected with cells treated with nEVs-210mimic compared to those injected with cells treated with nEVs-NC. The invasion rate was calculated as follows: invasion rate (%) = (the number of zebrafish with cell extravasated / the initial number of xenografted zebrafish) × 100. Values are expressed as mean ± SEM from 3 independent experiments

To further examinate the role of miR-210-3p in mediating tumor cell behavior in zebrafish xenograft model, we injected approximately 100–150 SK-N-AS cells treated with EVs into the Duct of Cuvier of each embryo expressing the green fluorescent vascular marker *Tg(fli1:EGFP)* at 48 hpf **(**Fig. 6A) [29]. Invasion was assessed through z-stack confocal images at 24 hpi. Cancer cells were identified as extravasated (cells found outside the vasculature) (Fig. 6B) and invaded (cells found into the avascular caudal tail region) (Fig. 6C). For the invasion rate, calculated as the number of zebrafish with cell extravasated/the initial number of xenografted zebrafish × 100, both cells extravasated and invaded were considered. Invasion at 24 hpi was observed in 68.7% of embryos injected with cells treated with nEVs-210mimic compared to 29.3% of those injected with treated with nEVs-NC (Fig. 6E).

Taken together these data indicate that cells pre-treated with EVs isolated from cells transfected with miR-210-3p-mimic and cultured in normoxic condition have a higher invasion ability in vivo compared to cells pre-treated with EVs isolated from cells transfected with miR-210-3p-NC. These results in the zebrafish xenograft model are consistent with the behavior of NB cells in vitro.

## Discussion

In Neuroblastoma, hypoxia, a key feature of solid tumors, has been linked to increased aggressiveness and to the presence of a larger subpopulation of tumor cells with stem cell-like properties [30]. Hypoxia also stimulates production of EVs, key messengers of biological signals that facilitate tumor intercellular communication [31]. Whether hypoxia of NB cells elicits signals that could be transmitted paracrinally and at a distance by EVs was unclear. Using a combination of in vitro and in vivo systems, we here show that EVs shed by hypoxic NB cells elicit transcriptional and functional changes in naïve target cells representing the primary tumor and its microenvironmental niches.

Whether and how EVs released by NB cell lines cultured in hypoxic conditions may contribute to tumor aggressiveness, progression and metastatic dissemination was unclear. Putative factors contributing to this role range from biophysical and structural differences, to changes in the EVs surface proteins, to differences in the biological responses elicited in the target cells. We therefore thoroughly characterized the morpho-functional features of EVs released by two NB cells lines that differ for their MYCN amplification status, and cultured in normoxia, hypoxia or hypoxia followed by reoxygenation. We did not find differences in EVs size and morphology, or in their surface markers presentation. The surface of all NB-derived EVs was decorated by proteins associated with cell adhesion and motility. We detected cell–cell adhesion molecules (CD146, CD326), and cell-to-extracellular matrix regulation molecules (CD29, CD44, CD49e). Their expression levels were not affected by the oxygenation conditions. NB-EVs also expressed receptor tyrosine kinase-like orphan receptor 1 (ROR1), a known inhibitor of apoptosis, potentiating EGFR signaling, and inducing EMT [32]. Importantly, ROR1 is detectable in embryonic tissue and generally absent in adult tissue, nominating it as an ideal anti-cancer drug target [33].

Despite the comparable expression of surface markers, EVs internalization in naïve SK-N-AS and SK-N-DZ cells was dependent on the oxygenation level of the parental cell culture. Specifically, h- and rEVs were more readily taken up than nEVs. Whether specific features of hEVs contribute to their enhanced uptake remains to be addressed in order to clarify the possible tissue specificity as well as their involvement in the formation of the premetastatic niche.

Activation of the EMT program predisposes cancer cells to acquire invasive and migratory properties [34]. Loss of E-cadherin, a major component of the adherens junctions, has long been considered a leading feature of EMT [35], together with the activation of core transcription factors including *SNAIL1, SNAIL2, ZEB1, ZEB2,* and *TWIST*, which repress genes encoding epithelial and activate those encoding mesenchymal characteristics. Indeed, hEVs stimulate migration and invasion of target SK-N-AS and SK-N-DZ naïve cells and correlates with changes in gene expression compatible with the activation of an EMT program. We show that hEVs activate EMT transcription factors like *TWIST, SNAIL* and *CDH1* which could explain the increased migration ability observed in target cells.

The exchange of information between the tumor microenvironment (TME) and tumor cells plays a key role in tumor progression. Recent reports suggested that EVs derived from the TME may communicate with cancer cells in the vicinity by transferring miRNA to regulate the metastatic potential of recipient cells [36–38]. miRNAs have generated great attention in oncology as they play a fundamental role in the regulation of gene expression, and their aberrant expression is present in almost all types of tumors, including pediatric ones. We here characterized the cargos of NB EVs identifying a total of 250 expressed miRNAs. Among them, we found that miR-210-3p was the most expressed in hEVs and persisted after reoxygenation. Therefore, we analyzed the expression level of miR-210-3p showing that miR-210-3p was highly expressed in cells cultured in hypoxic condition and their derived EVs, compared with their normoxic counterpart.

Hypoxia-regulated miR-210-3p contributes to important cell processes, including angiogenesis [39], DNA damage response [40], cell proliferation and apoptosis [41] through regulation of key gene targets such as E2F3 [42], TNIP1, SOCS1 [43], FGFRL1 [44], and STAT3 [45]. Hence, abnormal expression of miR‐210 can impact on cancer development [46, 47] and overexpression of miR‐210 has been associated with breast [48], renal [49], lung [50], colorectal [51] and pancreatic [52] cancers. However, the role of miR-210-3p in NB was still not clearly defined. We thus transfected NB cells with miR-210-3p mimic and inhibitor and purified EVs from these cells to investigate its effects in cancer metastasis. Initially, we confirmed the morphology of miR-210-3p overexpressed-EVs and the expression of the EVs markers tetraspanins CD81 and CD63, as well as the absence of negative marker Calnexin, by TEM analysis and immunoblot, respectively. We then questioned as to how EVs derived from transfected cells could influence the motility and invasiveness of SK-N-AS and SK-N-DZ cells and found an enhanced migratory and invasive ability of cells treated with EVs isolated from NB cells cultured in normoxia and transfected with miR-210-3p mimic. Furthermore, cells pre-treated with EVs isolated from cells transfected with miR-210-3p-inhibitor and cultured in hypoxic condition were apparently unable to migrate as efficiently as cells treated with hEVs, despite cells were cultured in hypoxic conditions. Our results verified that EVs miR-210-3p contributed to the pro-metastatic phenotype of NB cells in vitro, including enhanced migratory and invasive ability.

To determine whether the level of miR-210-3p in EVs released by NB cells affects cells migration and invasion in vivo, we established a xenograft model [53] using two zebrafish (Danio rerio) strains: the Casper strain lacking all melanocytes and iridophores [28] and the transgenic zebrafish line Tg(fli1:EGFP) expressing enhanced GFP in the vasculature [54]. The rapid and external development of transparent embryos [55], availability of reporter lines with traceable fluorescent cells [56], ease of genetic manipulation [57] and pharmacological approaches [58] make the zebrafish an excellent in vivo model to visualize single cell interactions in real time and to uncover the signaling mechanisms involved on a whole organism level [59]. First, to investigate the migration ability of cancer cells after treatment with EVs, we injected NB cells into the Yolk Sac of Casper embryos at 48 hpf and studied their intravasation and migration capacity throughout the vasculature. Although some cells could be observed in inter-somitic vessels, most NB cells arrested in the caudal vein plexus and SK-N-AS cells treated with nEVs-210mimic migrated from the yolk sac to the caudal vein plexus significantly more compared to hEVs-210inhibitor treated cells. Next, using the Tg(fli1:EGFP) transgenic line, we observed that miR-210-3p levels also influence the invasive ability of NB cells in vivo. We monitored the extravasation of fluorescently labelled NB cells by direct injection into the blood circulation (duct of Cuvier). Cancer cells were identified as extravasated (cells found outside the vasculature) and invaded (cells found into the avascular caudal tail region). We demonstrated that at 24 hpi the percentage of embryos with cells outside the vasculature, both extravasated and invaded, was higher in the group injected with cells pre-treated with nEVs-210mimic compared to the group injected with cells treated with nEVs-NC. Overall, these in vivo studies are consistent with the in vitro findings showing that miR-210-3p could promote tumor progression by transforming NB cells to a more aggressive phenotype.

## Conclusions

In conclusion, our findings suggest that EVs released by hypoxic tumor cells induce changes that favor a malignant phenotype in normoxic cells of the tumor and of the microenvironment by transferring miR-210-3p. Such changes range from increased migration and invasion properties to the expression of genes involved in EMT. This study represents the starting point for further investigations on the specific EVs-mediated biological processes favoring NB metastatic dissemination, both in vitro and in vivo. Ultimately, we expect that our approach might help to define potential biomarkers useful for early NB dissemination assessment and for the evaluation of tailored therapeutic strategies.

## Supplementary Information

**Additional file 1**: **Figure S1**. EVs characterization. A. Diameters of isolated EVs measured from TEM analysis. B. Representative EVs size distribution and concentration measured by qNano analysis. C. Representative EVs size distribution and concentration measured by nanoparticle tracking analysis. D. Background corrected APC median signal intensities for all 39 capture bead populations of the EVs surface from SK-N-AS and SK-N-DZ cells cultured in normoxia (grey bar), hypoxia (red bar), and reoxygenation (blue bar) conditions. Bars represent the mean ±SEM from at least three independent experiments. **Figure S2**. Uptake of NB-EVs. All experiments are performed on SK-N-AS cells treated with EVs from SK-N-AS cells cultured in normoxic, hypoxic and reoxygenation conditions. A. Uptake of NB-EVs by SKN-AS cells. Cells were cultured for 8 and 24 h in the presence of DiO-labelled EVs. Cells were stained with the Alexa Fluor 488 phalloidin (red) and DAPI (blue). Magnification 40X. Scale bar, 100 μm. B. Fluorescence intensity was evaluated in five random fields in sections using Image J. Graph represent values from at least n=3 independent experiments. *p<0.05, **p<0.01 and ****p<0.0001 vs normoxia. **Figure S3**. Heatmap shows a panel of 250 miRNAs with fluorescence intensity above the detection threshold (AU > 4.71) in each normoxia, hypoxia and reoxygenation condition. **Figure S4**. The workflow of miRNA filtering adopted comparing oxygenation condition between two cell lines is shown. **Figure S5**. Characterization of EVs from NB cells after transient transfection with miRNA-210-3p mimic and inhibitor. A. Transmission electron microscopy images of EVs isolated from cells transfected with miR210-inhibitor and miR210-mimic cultured in normoxic conditions. B. Validation of EVs markers expression by western blot analysis. C. Representative EVs size distribution and concentration measured by nanoparticle tracking analysis. **Figure S6**. EVs derived from transfected cells affected migration and invasiveness of SK-N-AS and SK-N-DZ cells. A. Representative images of wound healing assay performed on cells treated with EVs from transfected SK-N-AS and SK-N-DZ cells with miR-210-3p mimic; images were acquired at the time of EVs addition (T0) and 48 hours after treatment (T48H). Magnification 10X. Scale bar, 100 μm. Wound closure areas and cell velocity (μm/h) were quantified with Image J. Values are expressed as mean}SEM from 3 independent experiments. B Quantification of cells invaded toward the Matrigel layer (average of 5 picture fields at 10x magnification) were quantified with Image J. Values are means } SEM. C. Representative images of wound healing assay performed on cells treated with EVs from transfected SK-N-AS and SK-NDZ cells with miR-210-3p inhibitor; images were acquired at the time of EVs addition (T0) and 48 hours after treatment (T48H). Magnification 10X. Scale bar, 100 μm. Wound closure areas and cell velocity (μm/h) were quantified with Image J. Values are expressed as mean}SEM from 3 independent experiments. D. Quantification of cells migrated toward the Matrigel layer (average of 5 picture fields at 10x magnification) were quantified with Image J. Values are means} SEM.

**Additional file 2**: **Table S1**. Primer sequences used for quantitative PCR (qPCR).

**Additional file 3**: **Table S2**. NORMALIZED_DATA

## Data Availability

All original data are available at: https://researchdata.cab.unipd.it/id/eprint/818.

## Abbreviations

NB: Neuroblastoma
EVs: Extracellular vesicles
hEVs: Hypoxic extracellular vesicles
nEVs: Normoxic extracellular vesicles
rEVs: Reoxygenation extracellular vesicles
HIFs: Hypoxia-inducible factors
EMT: Epithelial-mesenchymal transition
TME: Tumor microenvironment
CM: Conditioned media
CTR: Negative control deprived of EVs
GO: Gene ontology
STRING: Search Tool for the Retrieval of Interacting Genes/Proteins
PCA: Principal component analysis
TEM: Transmission electron microscopy
qPCR: Quantitative PCR
hpf: Hours post fertilization
\hpi: Hours post injection
