# Supplementary material for: miR-210-3p enriched extracellular vesicles from hypoxic neuroblastoma cells stimulate migration and invasion of target cells
Source: Cell Biosci. 2023 May 18;13:89. doi: 10.1186/s13578-023-01045-z (PMC10193740; doi:10.1186/s13578-023-01045-z)
Supplement: Supplementary file 1 — Additional file 1: Figure S1. EVs characterization. A. Diameters of isolated EVs measured from TEM analysis. B. Representative EVs size distribution and concentration measured by qNano analysis. C. Representative EVs size distribution and concentration measured by nanoparticle tracking analysis. D. Background corrected APC median signal intensities for all 39 capture bead populations of the EVs surface from SK-N-AS and SK-N-DZ cells cultured in normoxia (grey bar), hypoxia (red bar), and reoxygenation (blue bar) conditions. Bars represent the mean ±SEM from at least three independent experiments. Figure S2. Uptake of NB-EVs. All experiments are performed on SK-N-AS cells treated with EVs from SK-N-AS cells cultured in normoxic, hypoxic and reoxygenation conditions. A. Uptake of NB-EVs by SKN-AS cells. Cells were cultured for 8 and 24 h in the presence of DiO-labelled EVs. Cells were stained with the Alexa Fluor 488 phalloidin (red) and DAPI (blue). Magnification 40X. Scale bar, 100 μm. B. Fluorescence intensity was evaluated in five random fields in sections using Image J. Graph represent values from at least n=3 independent experiments. *p<0.05, **p<0.01 and ****p<0.0001 vs normoxia. Figure S3. Heatmap shows a panel of 250 miRNAs with fluorescence intensity above the detection threshold (AU > 4.71) in each normoxia, hypoxia and reoxygenation condition. Figure S4. The workflow of miRNA filtering adopted comparing oxygenation condition between two cell lines is shown. Figure S5. Characterization of EVs from NB cells after transient transfection with miRNA-210-3p mimic and inhibitor. A. Transmission electron microscopy images of EVs isolated from cells transfected with miR210-inhibitor and miR210-mimic cultured in normoxic conditions. B. Validation of EVs markers expression by western blot analysis. C. Representative EVs size distribution and concentration measured by nanoparticle tracking analysis. Figure S6. EVs derived from transfected cells affected migration and [file 13578_2023_1045_MOESM1_ESM.pdf]

Extracellular vesicles from hypoxic neuroblastoma cells stimulate migration and invasion of target cells via miR-210-3p

Pina Fusco[1,2], Anna Fietta[1,2], Maria Rosaria Esposito[1,2], Sara Micheli[1], Angelica Bastianello[2], Lorenzo Bova[1], Luca Zanella[1], Giulia Borile[2], Giuseppe Germano[2] Elisa Cimetta[1,2*]

[1] University of Padua, Department of Industrial Engineering (DII) - Via Marzolo 9, 35131 Padova, Italy.

[2] Fondazione Istituto di Ricerca Pediatrica Città della Speranza (IRP) - Corso Stati Uniti 4, 35127 Padova, Italy.

[*]Corresponding author

e-mail address: elisa.cimetta@unipd.it (E. Cimetta)

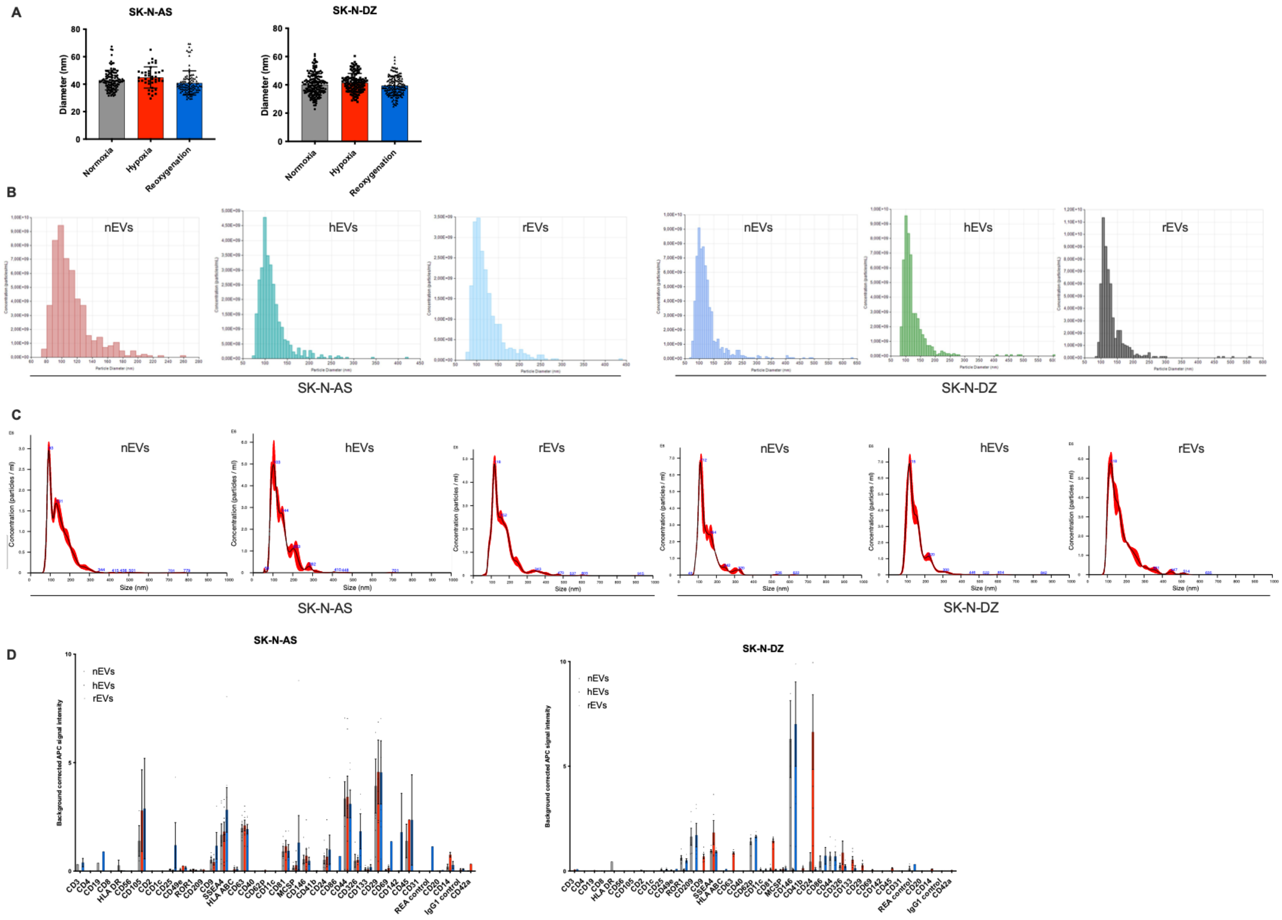

Figure S1. EVs characterization. A. Diameters of isolated EVs measured from TEM analysis**. B.** Representative EVs size distribution and concentration measured by qNano analysis. **C.** Representative EVs size distribution and concentration measured by nanoparticle tracking analysis. **D.** Background corrected APC median signal intensities for all 39 capture bead populations of the EVs surface from SK-N-AS and SK-N-DZ cells cultured in normoxia (grey bar), hypoxia (red bar), and reoxygenation (blue bar) conditions. Bars represent the mean ±SEM from at least three independent experiments.

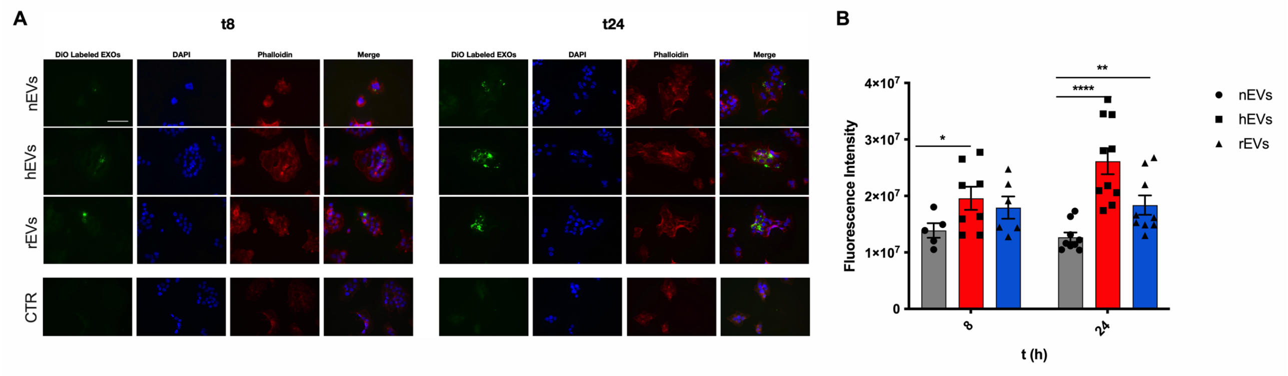

Figure S2. Uptake of NB-EVs. All experiments are performed on SK-N-AS cells treated with EVs from SK-N-AS cells cultured in normoxic, hypoxic and reoxygenation conditions. **A.** Uptake of NB-EVs by SK-N-AS cells. Cells were cultured for 8 and 24 h in the presence of DiO-labelled EVs. Cells were stained with the Alexa Fluor 488 phalloidin (red) and DAPI (blue). Magnification 40X. Scale bar, 100 µm. **B.** Fluorescence intensity was evaluated in five random fields in sections using Image J. Graph represent values from at least n=3 independent experiments. *p<0.05, **p<0.01 and ****p<0.0001 vs normoxia.

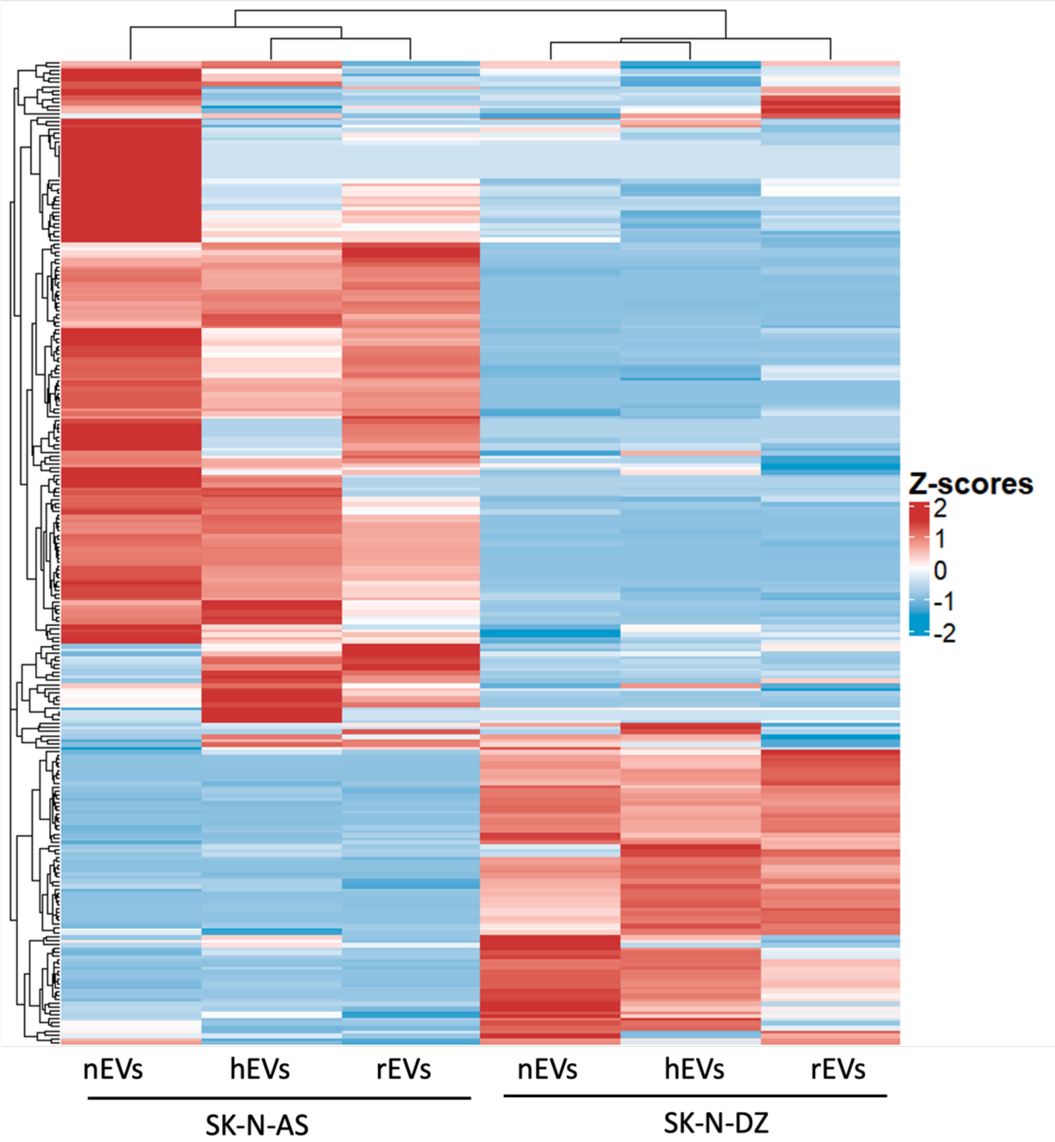

Figure S3. Heatmap shows a panel of 250 miRNAs with fluorescence intensity above the detection threshold (AU > 4.71) in each normoxia, hypoxia and reoxygenation condition.

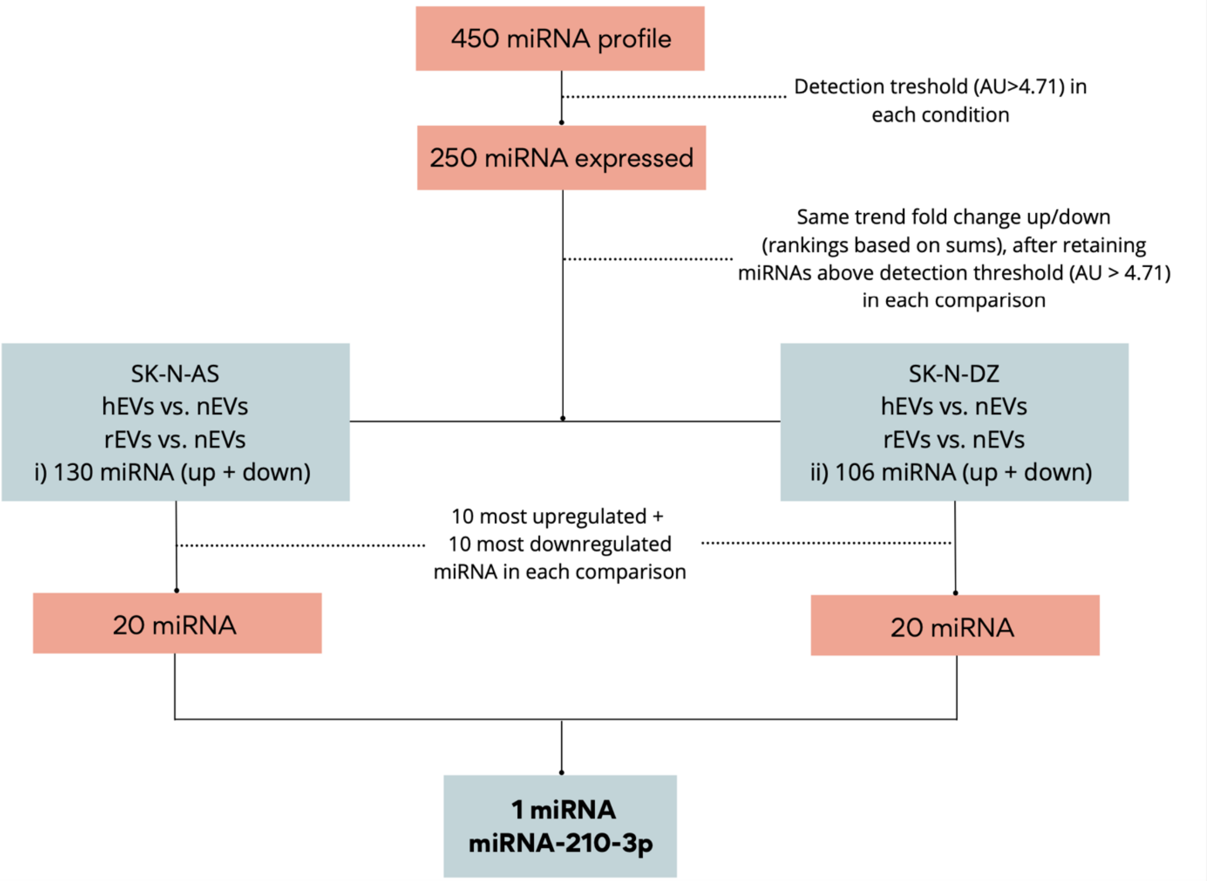

Figure S4. The workflow of miRNA filtering adopted comparing oxygenation condition between two cell lines is shown.

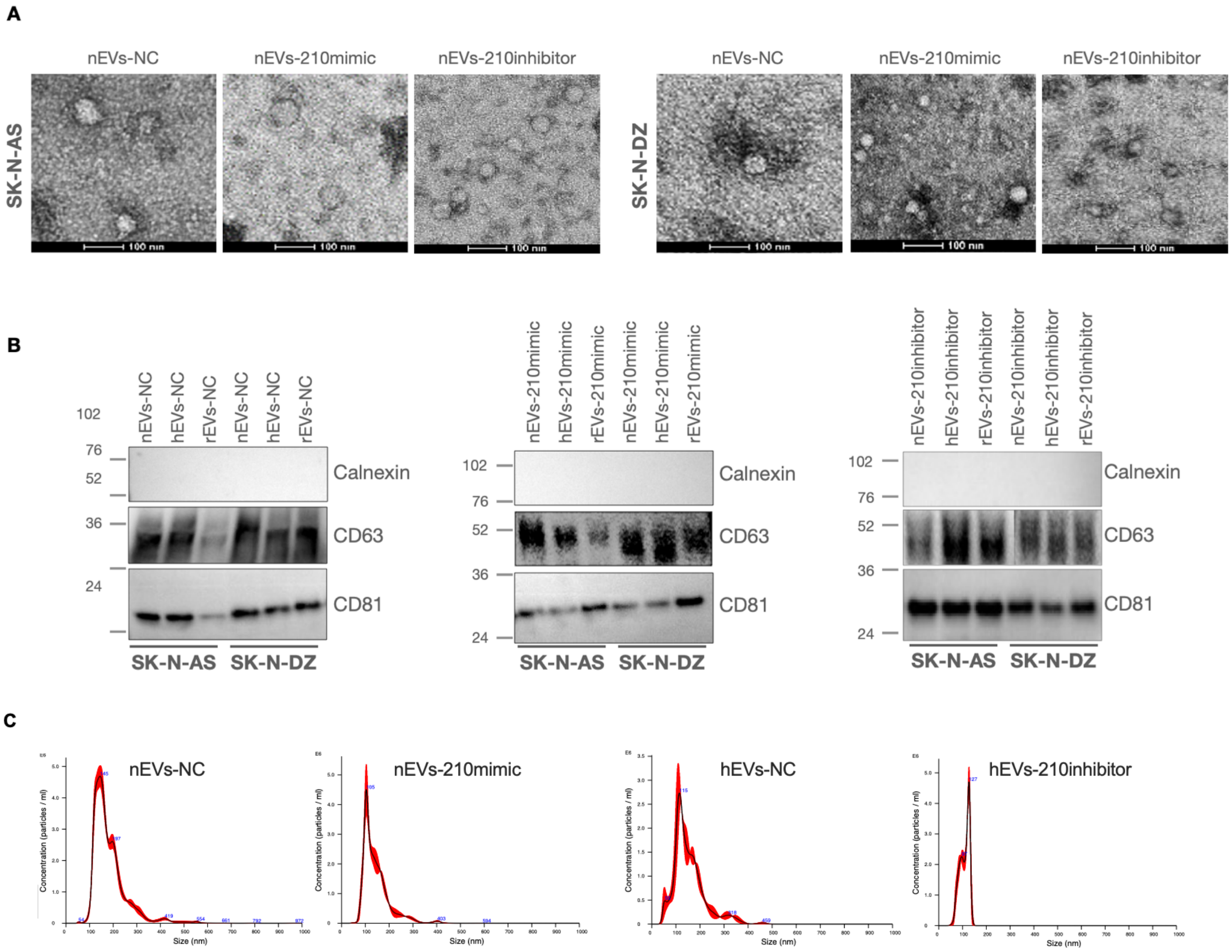

Figure S5. Characterization of EVs from NB cells after transient transfection with miRNA-210-3p mimic and inhibitor. A. Transmission electron microscopy images of EVs isolated from cells transfected with miR210-inhibitor and miR210-mimic cultured in normoxic conditions. **B.** Validation of EVs markers expression by western blot analysis. **C.** Representative EVs size distribution and concentration measured by nanoparticle tracking analysis.

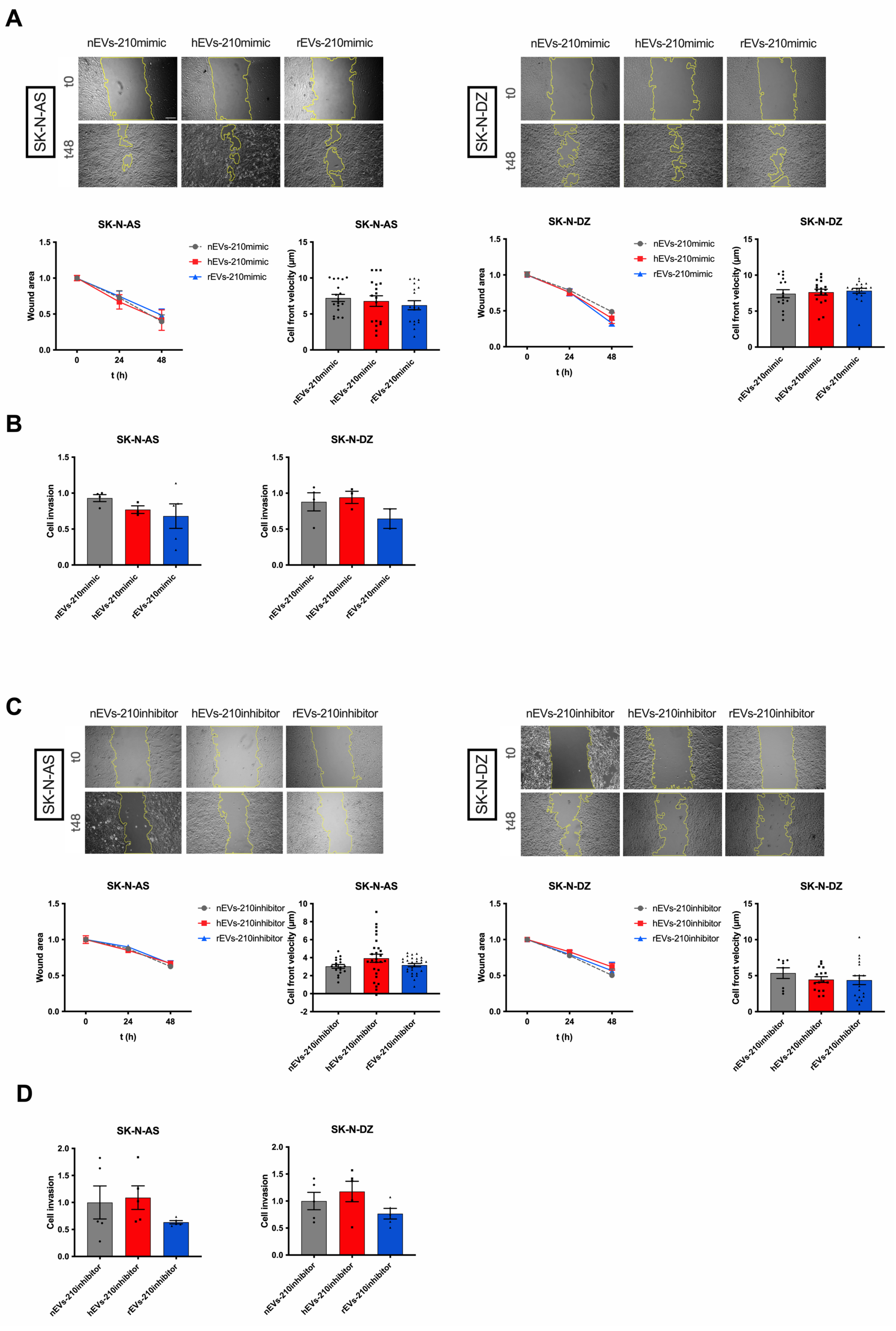
A
nEVs-210mimic
hEVs-210mimic
rEVs-210mimic
SK-N-AS
SK-N-DZ
t0
t48
Wound area
t (h)
Cell front velocity (µm)
B
Cell invasion
C
nEVs-210inhibitor
hEVs-210inhibitor
rEVs-210inhibitor
D

Figure S6. EVs derived from transfected cells affected migration and invasiveness of SK-N-AS and SK-N-DZ cells. A. Representative images of wound healing assay performed on cells treated with EVs from transfected SK-N-AS and SK-N-DZ cells with miR-210-3p mimic; images were acquired at the time of EVs addition (T0) and 48 hours after treatment (T48H). Magnification 10X. Scale bar, 100 μm. Wound closure areas and cell velocity (μm/h) were quantified with Image J. Values are expressed as mean±SEM from 3 independent experiments. **B** Quantification of cells invaded toward the Matrigel layer (average of 5 picture fields at 10x magnification) were quantified with Image J. Values are means ± SEM. **C.** Representative images of wound healing assay performed on cells treated with EVs from transfected SK-N-AS and SK-N-DZ cells with miR-210-3p inhibitor; images were acquired at the time of EVs addition (T0) and 48 hours after treatment (T48H). Magnification 10X. Scale bar, 100 μm. Wound closure areas and cell velocity (μm/h) were quantified with Image J. Values are expressed as mean±SEM from 3 independent experiments. **D.** Quantification of cells migrated toward the Matrigel layer (average of 5 picture fields at 10x magnification) were quantified with Image J. Values are means ± SEM.
